# Weighting Criteria and Prioritizing of Heat stress indices in surface mining using a Delphi Technique and Fuzzy AHP-TOPSIS Method

**DOI:** 10.1186/s40201-016-0264-9

**Published:** 2017-01-14

**Authors:** Mehdi Asghari, Parvin Nassiri, Mohammad Reza Monazzam, Farideh Golbabaei, Hossein Arabalibeik, Aliakbar Shamsipour, Armin Allahverdy

**Affiliations:** 1 Department of Occupational Health, School of Public Health, Tehran University of Medical Sciences, Tehran, Iran; 2 Department of Occupational Health, School of Public Health and Center for Air Pollution Research (CAPR), Institute for Environmental Research (IER), Tehran University of Medical Sciences, Tehran, Iran; 3 Research Center for Science and Technology in Medicine (RCSTIM), Tehran University of Medical Sciences, Tehran, Iran; 4 Department of Physical Geography, School of Geography, University of Tehran, Tehran, Iran; 5 Department of Biophysics & Biomedical Engineering, School of Medicine, Tehran University of Medical Sciences, Tehran, Iran

**Keywords:** Heat stress, Surface mining, Fuzzy AHP, Fuzzy TOPSIS

## Abstract

**Background:**

Heat stress as a physical harmful agent can increase the risk of health and safety problems in different workplaces such as mining. Although there are different indices to assess the heat stress imposed on workers, choosing the best index for a specific workplace is so important. Since various criteria affect an index applicability, extracting the most effective ones and determining their weights help to prioritize the existing indices and select the optimal index.

**Methods:**

In order to achieve this aim, present study compared some heat stress indices using effective methods. The viewpoints of occupational health experts and the qualitative Delphi methods were used to extract the most important criteria. Then, the weights of 11 selected criteria were determined by Fuzzy Analytic Hierarchy Process. Finally, fuzzy TOPSIS technique was applied for choosing the most suitable heat stress index.

**Results:**

According to result, simplicity, reliability, being low cost, and comprehensiveness were the most determinative criteria for a heat stress index. Based on these criteria and their weights, the existing indices were prioritized. Eventually, wet bulb glob temperature appropriated the first priority and it was proposed as an applicable index for evaluating the heat stress at outdoor hot environments such as surface mines.

**Conclusions:**

The use of these strong methods allows introducing the most simple, precise, and applicable tool for evaluation the heat stress in hot environments. It seems that WBGT acts as an appropriate index for assessing the heat stress in mining activities at outdoors.

## Background

Most workplaces depending on specific jobs and tasks may include the chemical, physical, biological, and ergonomic hazards that they cause a variety of effects ranging from minor injuries to serious diseases and even death. Heat is one of the most important physical hazards in many workplaces. Heat, as an energy source, exist in many industrial processes and workers who expose to it may be at risk of health problems and disorders [1]. Therfore, this adverse agent is an undeniable fact and considered as a major health problem around the world, especially in developing countries [2].

Heat stress is a combination of internal and external factors leading to heat related illnesses. The internal factors include internal body temperature, metabolic rate, and physiological adaptations to hot environments. The air temperature, thermal radiation, air velocity, and humidity contribute as the external factors [3]. Short-term exposure to extreme heat (acute exposure) can lead to rise the core body temperature, which it may directly cause heat related illnesses such as mild rash, cramps, heat exhaustion and heat stroke. It is reported that long-term chronic exposure to heat leads to chronic kidney diseases [4], cardiovascular diseases, and mental health problems [5].

In some industries, heat stress is a common problem by which workers continuously exposed to. These conditions are found in indoor workplaces such as ceramics, foundry, and glass and outdoor works like mining, agriculture and construction industries [6].

Mining activities lead to workersʼ exposure to undesirable environmental conditions. There are various causes for heat stress in mines. The sources of heat in surface mines include sun, electrical tools, machines, and mechanical processes. The efficiency of diesel engines typically is 33% and two-thirds of input energy is lost as heat, resulting in irreparable damages and disorders [7]. According to a study in New Zealand and India, miners are highly vulnerable to heat related illnesses than other workers, especially workers of indoor workplaces [6]. In 2013, an investigation assessing the sings and symptoms of heat-related illnesses showed 87% of workers in surface mines have experienced such symptoms, so that over 90% of them have occurred in warm seasons and over 80% happened more than once. This frequency of occurrences demonstrates the experiencing of heat-related symptoms is a common problem among workers of surface mines. High body temperature, headache, fatigue, and muscles cramps were the most common symptoms [8].

In Iran there are a lot of jobs and tasks with heat-related problems due to the weather conditions and the hot nature of most mining activities. According to the Statistical Centre of Iran in 2012, the total miners who worked in 6206 surface mines was 84528 [7]. Unfortunately, there is no statistics about miners exposed to heat stress and also no comprehensive study has been done to assess the heat stress and determine an index for mine workers. Therefore, it is necessary to determine an appropriate index to present the heat permissible limit due to geographical, environmental, and personal conditions.

There is a need for using the heat indices to assess the heat stress, protect the workers against extreme heat, and control the workplace heat stress. Based on literatures, since 1905 many efforts have been made to measure the levels of heat stress in workplaces and estimate the heat strain. The aim of presenting an suitable index is to specify the relationship between environmental parameters, clothing and activity as a number. Due to the complexity of variables related to heat stress in real working environment, determining a comprehensive heat stress index for each variable is maybe not possible. Differences in the physical work demands, health status, heat tolerance, and heat sources, as well as involvment of the heat-related mechanisms, job rotation, and environmental temperature may affect the risk levels [9]. Consequently, there have been over 60 heat stress indices indicating the scientific basis to establish the safety standards and limits for workers in hot environments. Heat indices are divided into 3 groups: logic indices based on heat balance equation, the experimental indices on the basis of objective and subjective measurement of heat strain, and direct indices based on the measurement of environmental variables [9].

The history of introducing and applying the heat stress indices is related to over a hundred years ago. The wet-bulb temperature index was firstly proposed by Haldane in 1905 as a measure to express the heat stress. After that, many indices were developed and applied around the world. Some of them include wet bulb glob temperature (WBGT), Effective temperature index, the Oxford index, 4- h Sweat Rate (P4SR), The new Universal Thermal Climate Index (UTCI). But none has been widely accepted [10].

This study was aimed to determine a heat stress index among the existing ones for sarface mines using a set of criteria. Considering the lack of certain and clear criteria for choosing a heat stress index, at first it was necessary to get the viewpoints of occupational health experts and use a qualitative method (Delphi) for defining some criteria to select an index.

Delphi is a method for acquiring the group knowledge. It has a structural process to predict and make decisions by a series of rounds, gather the information and eventually, achieve a group consensus [11]. Delphi is a systematic process to extract the experts opinions on a specified topic through a series of questionnaires. The anonymity of respondents and controlled feedback are essential [12]. Delphi is a simple, easy to use, and low cost tool that can be applied for gaining judgments on complex matters in lack of precise information.

The other advantages of the Delphi Method include high flexibility, elimination of the geographical impediments, providing open discussions, and focusing attention on the relevant issues [13]. Detailed planning and considering the effective components of Delphi study should be taken into account. Then, the weight of each criterion was determined by Fuzzy Analytic Hierarchy Process (AHP). Finally, using the fuzzy TOPSIS technique, a heat stress index was selected among the existing indices.

## Methods

This study is a qualitative practical research that was designed and implemented in 11 steps. Due to the fact that there were no common criteria for selecting a heat stress index, the Delphi technique was used.

This study was accomplished in following steps:

1. The formation of implementing team and monitoring the Delphi process
2. Selecting the experts and participants
3. Adjusting the questionnaire for first round
4. Editing the questionnaire grammatically (deductive and remove ambiguities)
5. Sending the questionnaire for experts
6. Analyzing the obtained responses in the first round
7. Preparing the second round questionnaire considering the required revisions
8. Sending the second questionnaire to the same experts
9. Analyzing the results of second questionnaire
10. Determining the relative weights of each criterion using the fuzzy AHP.
11. Choosing a heat stress index among the existing ones in the study using the fuzzy TOPSIS method.

A team including experts and consultants was formed and the aims of study were reviewed and revised. The number of 30 experts and panel members was selected from occupational health professionals of medical universities, administrative experts working in the department of environmental health in ministry of health, post-graduate students who were working on a thesis related to heat stress. According to studies, if the group is homogeneous, a sample consistent of ten to fifteen people will be sufficient for Delphi [14]. Therefore, selecting 30 participants in this study ensures the validity of findings in case of probable loss of respondants.

**Fig. 1:**
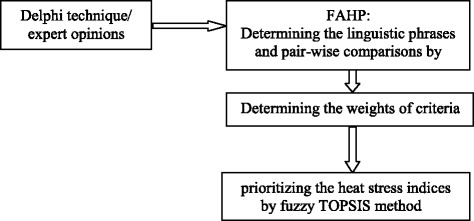
The conceptual model of the study

As a pilot test, an open question was designed and sent to 10 experts in order to survey their perceptions and response. Given the results of the pilot study, the questionnaire was sent to 30 mentioned participants. Then, the responses of first phase of study were analyzed, criteria extracted and prioritized. The criteria with less scores were also investigated. During several meetings with professors, some overlapped criteria with the same concept were merged. At the end of meetings, the criteria with the highest scores were extracted and used for prioritizing and pairwise comparing in the next step. The second questionnaire was designed and sent to the 30 previous members for pair-wise comparison between the criteria and finally determinig the weight of each criterion using Fuzzy AHP. For prioritizing the indices existing in the study using fuzzy TOPSIS, a questionnaire including *m* alternatives and *n* criteria was prepared and sent to the same experts. They were asked to assign a score for each alternative using the linguistic scale in order to select the most appropriate heat stress index for surface mines. Indices in this study were used with respect to their prevalence and validity, strong correlations with physiologic indices and other valid ones, and also their applicability for outdoor environments. Figure 1 indicates the conceptual model of the study.

### Fuzzy analytic hierarchy process

The Analytic Hierarchy Process (AHP) was proposed first time by Thomas. El. Saaty. This technique combines the opinions of all experts and provides the decision-making in a simple way. Then, a scale is used to pair-wise comparisons of criteria and assess their priorities [15]. This technique assays the quantitative and qualitative indices efficiently [16]. The advantages of this method include formulating the problem in question, improving the consistency of judgments, handling and solving the various problems, obtaining the opinions of members for making decision, aggregating the judgments of experts to determine the best alternative, and priotitizing through the pair-wise comparisons of criteria. The other advantage of AHP is the use of qualitative criteria for decision-making and expressing the results quantitatively by mathematical techniques. Using the quantitave results leads to intelligible data and accurate judgments [17]. the use of expert opinions based on the Delphi method ensures the acceptable results in the decision-making process. However, there are some disadvantages in the use of this method. For instance, AHP provides judgment based on the certain numbers without the reflecting of experience and knowledge of experts. To overcome this weakness, the combination of AHP and fuzzy logic and the use of fuzzy numbers are recommended. This approach can help resolve the weakness of criteria weighting [18]. After determining the criteria by Delphi technique, the following steps were performed to specify the weights of criteria using fuzzy AHP:

Determining the linguistic terms for pair-wise comparison the criteria [19] using Table 1.

**Table 1:** Membership function of linguistic scale

Linguistic	Fuzzy number	Scale of fuzzy number
Equal	1	(1, 1, 1)
Weak advantage	2	(1, 2, 3)
Not bad	3	(2, 3, 4)
Preferable	4	(3, 4, 5)
Good	5	(4, 5, 6)
Fairly good	6	(5, 6, 7)
Very good	7	(6, 7, 8)
Absolute	8	(7, 8, 9)
Perfect	9	(8, 9, 10)

Using the triangular fuzzy numbers to form the pair-wise comparison matrix.

**Fig. 2:**
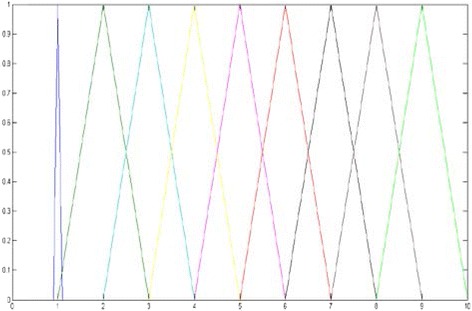
Triangular membership functions for linguistic values

In this study, to remove ambiguity of uncertainties in decision-making, the triangular fuzzy numbers presented in Table 1 were used in all steps for pair-wise comparisons in AHP. A triangular fuzzy number is composed of three factors, as Ã = (*l* + *m* + *u*), and its membership function is illustrated as follow:

Where, *m* is the maximum grade of membership function, *l* and *u* are the lower and upper bounds to allow more reasonable evaluation. A triangular fuzzy number (m, l, u) and the membership function μ (X) is expressed in Fig. 2. Matrix 2 shows the triangular membership function for linguistic terms [20].

1 $$ \upmu (x)=\left\{\begin{array}{l}\left(x-l\right)/\left(m-l\right),\kern1.8em x\in \left[l,m\right]\\ {}\left(u-x\right)/\left(u-m\right),\kern1.56em x\in \left[m,u\right]\\ {}\kern1.2em 0,\kern3.96em \mathrm{otherwise}\end{array}\right. $$

2 $$ \tilde{A}=\left[\begin{array}{l}1\kern0.6em \cdots \kern0.6em {\tilde{a}}_{1n}\\ {}\vdots \kern0.72em \ddots \kern0.96em \vdots \\ {}{\tilde{a}}_{n1}\cdots \kern0.72em 1\end{array}\right] $$

1. The use of fuzzy Geometric Mean [21].
  3 $$ {\tilde{r}}_i={\left({\tilde{a}}_{i1}\otimes {\tilde{a}}_{i2}\otimes \dots \otimes {\tilde{a}}_{in}\right)}^{\raisebox{1ex}{$1$}\!\left/ \!\raisebox{-1ex}{$n$}\right.} $$
2. Calculating the fuzzy weight of each elements.
  4 $$ {\tilde{w}}_i={\tilde{r}}_1\otimes {\left({\tilde{r}}_1\oplus {\tilde{r}}_1\oplus \dots \oplus {\tilde{r}}_1\right)}^{-1} $$
3. The use of best non fuzzy performance (BNP)
  5 $$ BNPi=\raisebox{1ex}{$\left[\left(\mathrm{u}\mathrm{i}-l\mathrm{i}\right)+\left(\mathrm{m}\mathrm{i}-l\mathrm{i}\right)\right]$}\!\left/ \!\raisebox{-1ex}{$3$}\right.+li $$
4. Calculating the inconsistency rate of matrix
  The subjective comparisons in this method leads to some inconsistencies in AHP. The consistency ratio (CR) can be calculated as follow. If the CR is unacceptable, pair-wise comparison should be reconsidered.
  6 $$ CR=\frac{CI}{RI} $$

The consistency index (CI) presents the deviation from the consistency and is calculated as follow:

7 $$ CI=\frac{\lambda_{\max }-n}{n-1} $$

where, *n* is the size of pair-wise comparison matrix, λ_*max*_ is the maximal value of comparison matrix, and RI is the random consistency index obtained randomly from related tables [15]. If CR < 0.1, the comparisons are acceptable. The CR > 0.1 indicates the inconsistent judgments and pair-wise comparison should be revised [22].

The TOPSIS is a multi criteria decision-making method introduced firstly by Hwang and Yoon. The basic concept of TOPSIS is providing an ideal solution to maximize the benefit criteria and minimize the cost criteria. Briefly, the positive ideal solution includes the best available criteria while the negative ideal solution is consistent of the worst values of criteria. Therefore, optimized alternative has the least distance from the positive ideal solution and the farthest distance from the negative one. The TOPSIS algorithm is a strong compensatory multi-criteria decision-making technique to prioritize the alternatives with regard to the ideal solution. It has a little sensitivity to type of weighting technique and its responses don’t significantly change. In addition, an advantage of this model is its ability for fast identifying the best alternative. In this algorithm, it is assumed that each index and criterion in decision-making increases or decreases uniformly [23].

It is often difficult for decision-makers to present an precise value for criteria due to some errors in this respect. In this case, it is recommended the use of fuzzy numbers for assessments. Therfore, in this study the fuzzy TOPSIS was applied for prioritization. The steps of fuzzy TOPSIS are as follows:

Step 1. Constructing the initial comparison matrix. The fuzzy values in this matrix have been defined in the scale of membership functions in Table 2.

**Table 2:** Linguistic variables for ratings

Linguistic variable	Fuzzy number
Very poor (VP)	(0, 0, 1)
Poor (P)	(0, 1, 3)
Medium poor (MP)	(1, 3, 5)
Fair (F)	(3, 5, 7)
Medium good (MG)	(5, 7, 9)
Good (G)	(7, 9, 10)
Very good (VG)	(9, 10, 10)

Step 2. Determining the positive (benefits) and negative (costs) ideal solutions as A+ and A- respectively.

$$ \begin{array}{l}{A}^{+}=\left({\tilde{p}}_1^{+},{\tilde{p}}_2^{+},\dots, {\tilde{p}}_m^{+}\right)\\ {}{A}^{-}=\left({\tilde{p}}_1^{-},{\tilde{p}}_2^{-},\dots, {\tilde{p}}_m^{-}\right)\end{array} $$

where:

$$ \begin{array}{l}{\tilde{p}}_j^{+}=\left( \max {\tilde{p}}_{ij},j\in {J}_1;\underset{i}{ \min }{\tilde{p}}_{ij},j\in {J}_2\right)\\ {}{\tilde{p}}_j^{-}=\left(\underset{i}{ \min }{\tilde{p}}_{ij},j\in {J}_1;\underset{i}{ \max }{\tilde{p}}_{ij},j\in {J}_2\right)\end{array} $$

where J_1_ and J_2_ respectively present the criteria benefit and cost.

Step 3. Calculating the distance of each alternative from negative and positive ideal solutions by Euclidian (n-dimension) method.

$$ \begin{array}{l}{d}_i^{+}={\displaystyle \sum_{j=1}^nd\left({\tilde{p}}_{ij},{\tilde{p}}_j^{+}\right),\mathrm{with}\kern0.24em i}=1,\dots, m.\\ {}{d}_i^{-}={\displaystyle \sum_{j=1}^nd\left({\tilde{p}}_{ij},{\tilde{p}}_j^{-}\right),\mathrm{with}\kern0.24em i}=1,\dots, m.\end{array} $$

Where *di* ^+^ *and d* ^−^ represents the distance of alternative from positive and negative ideals. Also, distance (*d*) between two fuzzy numbers (*Pij*, *Pi* ^+^) is calculated by following equation:

$$ d\left(\tilde{a},\tilde{b}\right)=\sqrt{\frac{1}{3}\left[{\left({a}_1-{b}_1\right)}^2+{\left({a}_2-{b}_2\right)}^2+{\left({a}_3-{b}_3\right)}^2\right]}. $$

Step 4. Calculating the relative closeness of each alternative to the best solution as follow:

$$ \xi {}_i=\frac{d_i^{-}}{d_i^{+}+{d}_i^{-}}. $$

Step 5. Prioritizing the alternatives using the relative closeness. Each alternative which has the largest closeness is the best for choosing.

## Results

The aim of this study was to determine the effective criteria for choosing a heat stress index by using the Delphi model and specify those criteria weights by fuzzy AHP. Using 30 completed questionnaires by experts and reviewing their viewpoints, 30 criteria were extracted. In another meeting, due to the same concepts of some criteria and the lack of relevance of some of them, the effective criteria to select the index decreased to 14. Finally, 11 criteria were selected considering the percentage of experts responses. They can be seen in Table 3. According to the table, the Simplicity criterion by 76.6% appropriated the highest percentage of responses.

**Table 3:** The extracted criteria to determine a heat stress index

No.	Extracted criteria	Frequency	Percent
1	Simplicity	23	76.6
2	Reliability	16	53.3
3	Low cost	16	53.3
4	Comprehensiveness	15	50
5	Direct reading	14	46.6
6	Precision	14	46.6
7	Strong correlation with the physiological strain indices	13	43.3
8	Non-interferencing with worker activity, the work process, and quality of work	10	33.3
9	Availability	8	26.6
10	being influenced by other factors	7	23.3
11	Being standard	7	23.3

As shown in Table 2, in this study 11 criteria were considered with regard to problem in question. Then, the selected criteria with high agreement were sent back to experts for pair-wise comparisons. Finally, the weight of each criterion was analyzed by fuzzy AHP considering the importance of criteria toward the interested aims. The consistency rate (CR) of this study was 0.083, lower than 0.1, that is acceptable. According to Table 4, the criterion of “being standard” had the highest relative weight (0.141). The relative weights (BNP) of other criteria can be seen in Table 3.

**Table 4:** The coefficients of evaluation matrices and the weighted vector of criteria

No.	Extracted criteria	Smallest expected value (*l*)	Most probable expected value (*m*)	Largest expected value (*u*)	Relative weight (BNP)
1	Simplicity	0.037	0.041	0.045	0.041
2	Reliability	0.111	0.130	0.143	0.128
3	Low cost	0.021	0.032	0.044	0.032
4	Comprehensiveness	0.058	0.090	0.128	0.092
5	Direct reading	0.042	0.060	0.069	0.057
6	Precision	0.121	0.142	0.157	0.140
7	Strong correlation with the physiological strain indices	0.101	0.109	0.126	0.112
8	Non-interferencing with worker activity, the work process, and quality of work	0.085	0.089	0.102	0.092
9	Availability	0.06	0.062	0.068	0.063
10	being unaffected by other factors	0.081	0.109	0.116	0.102
11	Being standard	0.128	0.146	0.149	0.141

**Fig. 3:**
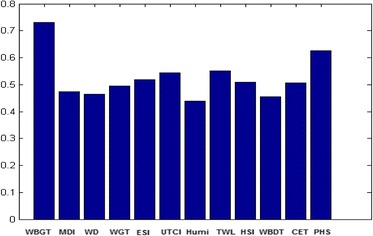
Ranking the heat stress indices using the fuzzy TOPSIS

Results related to the closeness factor (the distanse from positive and negative ideal solutions) and prioritization of heat stress indices using fuzzy TOPSIS technique are seen in Table 5. According to findings, the first priority for surface mines was the WBGT index with closeness factor of 0.730. The normalized fuzzy decision-making matrix has beeb presented in Table 6. Figure 3 indicates the prioritizing (ranking) of heat stress indices. All criteria of this study were positive.

**Table 5:** Proritization and comparison of the heat stress indices in this study

Heat Stress Index	Ranking	Positive ideal solution	Negative ideal solution	Relative closeness
Wet Bulb Globe Temperature (WBGT)	1	0.221	0.598	0.730753
Modified discomfort index (MDI)	9	0.438	0.389	0.47329
Oxford index (WD)	10	0.445	0.381	0.463475
Wet Globe Temperature (WGT)	8	0.421	0.405	0.493591
Environmental Stress Index (ESI)	5	0.401	0.426	0.517439
Universal Thermal Climate Index (UTCI)	4	0.380	0.446	0.543306
Humidex	12	0.468	0.360	0.439264
Thermal work limit (TWL)	3	0.375	0.451	0.551512
Heat Stress Index (HSI)	6	0.408	0.420	0.509846
Wet-bulb dry temperature (WBDT)	11	0.454	0.374	0.454052
Corrected Effective temperature (CET)	7	0.410	0.418	0.506904
Predicted Heat Strain (PHS)	2	0.309	0.515	0.625184

**Table 6:** The normalized fuzzy decision-making matrix using triangular fuzzy number (C1-C11: The criteria number 1 to 11)

The lower limit											
	C1	C2	C3	C4	C5	C6	C7	C8	C9	C10	C11
WBGT	0.030	0.087	0.023	0.053	0.039	0.084	0.076	0.068	0.045	0.048	0.118
MDI	0.031	0.049	0.025	0.025	0.024	0.051	0.036	0.068	0.046	0.037	0.039
WD	0.031	0.049	0.025	0.023	0.024	0.051	0.035	0.068	0.044	0.036	0.037
WGT	0.031	0.053	0.022	0.026	0.033	0.051	0.042	0.065	0.039	0.039	0.045
ESI	0.014	0.073	0.013	0.042	0.015	0.073	0.055	0.053	0.023	0.046	0.053
UTCI	0.014	0.073	0.013	0.045	0.016	0.085	0.058	0.054	0.019	0.050	0.057
Humidex	0.024	0.048	0.020	0.028	0.018	0.050	0.037	0.061	0.034	0.036	0.036
TWL	0.016	0.074	0.015	0.044	0.017	0.075	0.063	0.052	0.024	0.048	0.058
HSI	0.012	0.072	0.013	0.043	0.011	0.068	0.067	0.040	0.020	0.045	0.062
WBDT	0.031	0.052	0.024	0.022	0.023	0.048	0.035	0.066	0.042	0.032	0.037
CET	0.024	0.063	0.021	0.030	0.019	0.064	0.048	0.063	0.034	0.038	0.046
PHS	0.010	0.089	0.011	0.057	0.013	0.093	0.074	0.040	0.017	0.061	0.106
The median limit											
	C1	C2	C3	C4	C5	C6	C7	C8	C9	C10	C11
WBGT	0.036	0.110	0.028	0.068	0.046	0.110	0.094	0.080	0.054	0.066	0.137
MDI	0.038	0.073	0.030	0.038	0.032	0.076	0.055	0.081	0.055	0.054	0.058
WD	0.037	0.072	0.030	0.036	0.032	0.076	0.053	0.081	0.054	0.052	0.056
WGT	0.037	0.077	0.027	0.041	0.041	0.075	0.061	0.078	0.049	0.056	0.065
ESI	0.021	0.098	0.019	0.057	0.022	0.099	0.075	0.068	0.033	0.064	0.074
UTCI	0.020	0.098	0.019	0.060	0.023	0.111	0.079	0.069	0.029	0.069	0.078
Humidex	0.031	0.072	0.026	0.042	0.027	0.074	0.055	0.075	0.044	0.053	0.056
TWL	0.023	0.099	0.021	0.059	0.025	0.101	0.083	0.067	0.035	0.067	0.080
HSI	0.019	0.096	0.019	0.059	0.018	0.094	0.085	0.055	0.030	0.064	0.084
WBDT	0.037	0.076	0.030	0.036	0.030	0.073	0.054	0.079	0.052	0.049	0.056
CET	0.031	0.087	0.027	0.046	0.027	0.089	0.068	0.077	0.045	0.056	0.067
PHS	0.016	0.112	0.016	0.072	0.020	0.118	0.091	0.055	0.027	0.079	0.127
The upper limit											
	C1	C2	C3	C4	C5	C6	C7	C8	C9	C10	C11
WBGT	0.040	0.127	0.035	0.080	0.051	0.130	0.105	0.089	0.061	0.082	0.143
MDI	0.041	0.097	0.036	0.054	0.039	0.101	0.074	0.088	0.062	0.071	0.081
WD	0.040	0.096	0.036	0.052	0.042	0.101	0.079	0.088	0.063	0.070	0.080
WGT	0.040	0.101	0.033	0.056	0.048	0.100	0.081	0.087	0.055	0.074	0.098
ESI	0.028	0.119	0.026	0.072	0.031	0.122	0.093	0.080	0.043	0.081	0.098
UTCI	0.027	0.118	0.026	0.074	0.031	0.130	0.095	0.080	0.039	0.086	0.100
Humidex	0.036	0.096	0.032	0.058	0.035	0.099	0.078	0.083	0.053	0.072	0.079
TWL	0.030	0.119	0.028	0.074	0.033	0.125	0.099	0.078	0.047	0.085	0.103
HSI	0.026	0.117	0.029	0.074	0.026	0.120	0.099	0.069	0.041	0.082	0.106
WBDT	0.041	0.100	0.043	0.052	0.041	0.097	0.075	0.087	0.059	0.067	0.081
CET	0.042	0109	0.037	0.062	0.039	0.115	0.087	0.085	0.054	0.074	0.092
PHS	0.024	0.127	0.023	0.081	0.028	0.135	0.103	0.070	0.037	0.092	0.139

## Discussion

Heat stress is a hazardous physical agent in the workplaces. Nowadays, due to the technological advances, climate change, and consequently the increasing of global temperature, the heat stress is considered as a adverse factor in many workplaces. Exposure to heat causes heat syncope, heat exhaustion, heat stroke, confusion, poor concentration, and also exacerbation of some diseases, as well as reducing the production and increasing the indirect costs. Particularly, long-term exposure to hot conditions can result in so much stress on individuals [24]. The heat stress indices are used to assess this hazardous agent. These indices summarize the existing environmental conditions and express it as a number. In spite of many indices for assessment of heat stress and their advantages and disadvantages, there is no acceptable global comprehensive index for that. Therefore, it is necessary determining effective criteria to choose the most appropriate and precise heat stress index among existing ones [10]. In this study, the effective criteria for selecting a heat stress index were investigated. A group of the most important criteria, determined using the Delphi method and experts viewpoints, were simplicity, reliability, comprehensiveness, being direct reading, precision, strong correlation with the physiological strain indices, and so on. At first, the experts were asked to list the criteria affected on the selecting a heat stress index in order to extract the significant criteria. After some meetings with experts and omitting a number of criteria, 11 of 30 criteria were selected (Table 2). The extracted criteria were next sent to experts to determine the importance of each of them by pair-wise comparisons. In the other word, using the pair-wise comparisons and fuzzy AHP was resulted in specifying the real weights of criteria.

Among the obtained responses, the simplicity criterion had the highest frequency. Simplicity of an index means the calculations are feasible without complex computing and multiple devices. Although this criterion appropriated the highest percentage of responses (76.6%), its relative weight was 0.041 (the least weight after the criterion of “being low-cost”) and had the less importance in comparison with other criteria. The next criterion with the most frequency was the reliability of index. Reliability refers to the fact that if the measurements are repeated several times in the same weather conditions, the similar results will obtain. The percentage of responses was 53.3%. The percentage for “being low-cost” the heat stress index was the same as the reliability (53.3%). The criteria of comprehensiveness, being direct reading, and precision were located in the next priorities with the percentage of 50, 50, and 46.6%, respectively. The Fuzzy analytic hierarchy process (AHP) showed that according to the opinions of experts, the maximum weight was related to “being standard” of index. This criterion means that if the index is acceptable by international organizations or not? For instance, The International Organization for Standardization (ISO) publish the standards in many fields [10]. The criteria of precision, reliability, strong correlation with the physiological strain indices, and being unaffected by other factors had the higher weights, respectively (the priorities of secend to fifth). Although there is no study to determine the effective criterion for choosing a heat stress index, the National Institute for Occupational Safety and Health (NIOSH) has proposed some criteria for using an index. They are as follow:

1. It should be applicable and precise when using;
2. It should be consisted of all important factors (environmental, metabolism, and clothing ones);
3. Its measurements and calculations should be simple;
4. The means and methods used to measure the workers’ exposure should not interfere with their activities;
5. The index should be applicable in the wide range of environmental and metabolic conditions [25].

It is worth mentioning that, the NIOSH recommendations to choose an appropriate index is aligned with the results of this study. So it can be concluded that to have a reliable and accurate measurements, the first step is choosing a suitable and precise index. To select an index based on the viewpoints of experts, its being standard is the most important criterion. The precision and reliability of index, and its strong correlation with the physiological strain indices should be considered. In this study for choosing an index among existing ones, the fuzzy TOPSIS technique was used. The TOPSIS algorithm is one of the most reliable scientific and management methods for decision-making. Using this technique, with regards to all effective aspects, it is possible assessing the criteria types, priorities, and weights toward each other, evaluating the indices relative to the criteria, and also prioritizing the indices in a resonable way. The results of closeness factor and indices priorities can be seen in Table 5. The results showed that for surface mines the WBGT index by the closeness factor of 0.730 appropriated the first priority among the existing indices. There are some important reasons for this result. This index by using the natural wet bulb temperature, globe and air temperatures can express the thermal conditions numerically. In addition to simplicity, WBGT has the high efficiency for assessing the thermal conditions. It is able to evalute the effects of heat during a period of time in which one performs an individual activity. It should be noted that the WBGT is the most widely used index around the world. By this index, there is an acceptable relationship with the physiological parameters at high temperatures [26]. The World Health Organization (WHO), International Organization for Standardization (ISO), and the National Institute of Occupational Safety and Health (NIOSH) have also recommended the WBGT as an index for assessing the heat stress. The Standard ISO-7247 has been introduced by ISO for measurement and evaluation of heat stress [10]. Alfano in 2014 conducted a review study on revision the WBGT index after 60 years of use.

It is said that, the WBGT index is simple to understand and use, and also it has suitable validity to organize the work–rest cycle. The fact that, there are some limitations related to WBGT and they would be preferable to consider for protecting the workers who work in hot environments [27]. According to Parsons (2006), the WBGT index, accepted by ISO, has adequate validity, releability, and applicability to control the heat stress in military, industrial, sports and commercial places around the world [10].

Based on Table 5, among the heat stress indices, the Predicted Heat Strain index (PHS) had the second priority with the closeness factor of 0.625. The PHS, the resultant of modifying the required sweat rate (SWreq) index, was proposed by ISO-7933 standard in 2004 [28]. This index was developed by a team of European researchers [29]. The PHS predicts the sweat rate and the internal core temperature for workers in hot environments. Although the PHS is not able to predict the response of each worker, it can predict the heat stress conditions by which the body core temperature increases. Consequently, it can estimate the maximum allowable exposure duration for work in hot workplaces. Because of complex calculations, this index is computed by a software. The required factors include air temperature, wet temperature, radiant temperature, relative humidity, air velocity, metabolic rate, partial vapor pressure, and thermal resistance of clothing [30]. Based on the fuzzy AHP and experts’ viewpoints, among the criteria the highest weight was obtained for the “being standard” of predicted heat strain index. Being standard means that an index has been accepted by international organizations. It is consistent with the results of this study, because the WBGT and PHS indices have been recommended by ISO [27].

Holmer in 2010 conducted an investigation using internationally proposed methods including WBGT-ISO 7243 and PHS-ISO 7933. The results obtained from the comparative evaluation of heat stress at the same conditions with PHS and WBGT showed the WBGT index has more conservative assessment approach compared to PHS, resulting in much shorter working time. It seems that, WBGT overestimates the heat stress. This is aligned with findings related to some developing countries in which work is done under climatic conditions. In this case, considerable limitations are necessary when following the WBGT recommendations. Although, considering the data from warm countries, the predicted physiological strain by PHS may be more appropriate, the conservative philosophy of WBGT can be valuable in regard to safety [31]. Among the indices, the third priority was related to Thermal Work Limit (TWL) index with closeness factor of 0.551. It was proposed by Bates and Brake in 1997. The allowable limits of this index were firstly provided for underground industries (2002), and then these limits were proved for surface workplaces by Miller and Bates [32]. The TWL is a rational heat stress index that offers maximum tolerable rate of metabolism. So that, acclimatized person with suitable hydration status can work in the specific hot environment, provided that the body core temperature is less than 38.5 °C and the sweat rate is less than 1.2 kg/h. This index is based on the experimental studies of human heat transfer and humidity and heat tranfer equations through clothing. The parameters of clothing are variable and this protocol can be used for unacclimatized people [33]. As an advantage of this index, there is no need for estimating the actual metabolic rate. The aim of the index is the calculating the maximum metabolic rate (W/m^2^) that it can be continuously tolerated in hot environments. The maximum level of work in hot environments is calculated by measuring dry, wet, and radiation temperatures, wind speed, and atmospheric pressure, and also by considering the adaptability status and type of clothing. Also the safe work duration and guidelines for work-rest cycle in hot environments can be determined by this index. It is able to calculate the sweat rate and determine the needs for fluid intak for preventing dehydration. This index has taken the code of practice from the Abu Dhabi management system of health safety and environment to manage the heat stress [33].

Table 5 illustrates the forth priority of Universal Thermal Climate Index (UTCI) index with closeness factor of 0.543. As a new heat stress index for outdoor hot environments, the UTCI was developed in 2011 by international institute of biometeorology. It was extended by COST (a European Union program promoting Cooperation in Science and Technology) Action 730 [34]. It was derived from the scientific advances in the fields of human thermal physiology, biophysic, and heat transfer theories. It was proposed for developing appropriate standards to assess the heat stress in outdoor environments. Compared to other indices, it is sensitive to small changes in air temperature, wind, radiation, and humidity and it can be used in various climatic conditions [35]. Therefore, the UTCI index provides an appropriate assessment for impacts of climate changes on human. This index categorizes the thermal stresses in 10 classes, from extreme cold stress to extreme heat stress, as an equivalent temperature between −50 and +50 [36].

Among the indices, Environmental Stress Index (ESI) with the closeness coefficient of 0.517 had the fifth priority. Moran et al. (2001) proposed ESI based on the measurement of air temperature, relative humidity (RH), and solar radiation (SR). These three variables are commonly used due to the quick response and simple measurement. Also, they can be determined using commercially available fast-reading sensors [37]. In addition to a high correlation between the ESI and WBGT index, the ESI correlation with the physiological parameters (core temperature, heart rate, and sweat rate) was considerable [38]. Following the assessment under various thermal conditions, ESI was proposed as a good alternative index for WBGT at the same conditions [39].

According to the results of studies conducted in mines of different countries, the WBGT, TWL, and effective temperature indices have been used seperately or in combination with each other, that it is consistent with selected indices in present study [40]. A study by Naghib indicated good correlation between WBGT and physiological parameters in surface mines, while this relationship was weak for undergroud mines [41].

To determine the optimal heat stress indices, Omidvari et al. evaluated the SWreq, WBGT, P4SR, and CET in surface mines located in Kermanshah province of Iran. In this respect, the criteria of heart rate and skin and core temperatures were also considered. The obtained results recommended WBGT and SWreq as the most appropriate indices to assess the heat stress [42]. Golbabaei et al. compared the indices DI, WBGT and SWreq in an outdoor industry with warm and humid conditions. Their investigation illustrated the WBGT index had the highest correlation with heart rate and the most optimal value compared to other indices. It was recommended for fast assessment of thermal stress in such thermal conditions, WBGT is often more valid index than the other indices [43]. Brake et al. (2002) compared different indices of heat stress to determine the best one. It was found that although there is considerable difference between the allowable limits of various indices, WBGT is one of the most appropriate indices to evaluate the thermal stress [44].

Since the activities in surface mines are performed under the sunlights, as a heat source, it can be concluded that in such conditions the WBGT index is more suitable than other indices and indicates the actual heat stress imposed by the environment. A study by Brief found that the WBGT index acts more accuratly than other indices in presence of radiation energy in the environment and it can indicated the actual thermal conditions. These findings are in line with results obtained in the present study.

## Conclusion

In conclusion, this study demonstrates that WBGT is often the most appropriate index to evaluate the heat stress in surface mines. Some criteria may affect prioritizing the heat stress indices and choosing a suitable index among them. In this respect, the most important criteria can be extracted using experts’ viewpoints and Delphi method. Simplicity, reliability, being low cost, and comprehensiveness are determinative criteria for applicability of an index. Determining the weights of criteria and prioritizing the indices are performed by the fuzzy AHP and TOPSIS techniques, respectively. The use of these strong methods allows introducing the most simple, precise, and applicable tool for evaluation the heat stress in hot environments. It seems that WBGT acts as an appropriate index for assessing the heat stress in mining activities at outdoors.

## Abbreviations

BNP: Best non fuzzy performance
CET: Corrected Effective temperature
CI: Consistency index
CR: Consistency ratio
ESI: Environmental Stress Index
FAHP: Fuzzy Analytic Hierarchy Process
HSI: Heat Stress Index
ISO: The International Organization for Standardization
MDI: Modified discomfort index
PHS: Predicted Heat Strain index
RH: Relative humidity
SWreq: Required sweat rate
TOPSIS: Technique for Order Preference by Similarity to Ideal Solution
TWL: Thermal Work Limit
WBDT: Wet-bulb dry temperature
WBGT: Wet bulb glob temperature
WD: Oxford index
WGT: Wet Globe Temperature
